# Asphalt VOC Emission Reduction Mechanism Based on Molecular Simulation and Structural Regulation of Zeolites

**DOI:** 10.3390/ma19132753

**Published:** 2026-06-28

**Authors:** Jia Guo, Qiang Li, Yimeng Lei, Xiwen Chang, Yue Xiao, Mohammed H. Al Mehthel, Yufei Zhang

**Affiliations:** 1 School of Materials Science and Engineering, Chang’an University, Xi’an 710064, China; guojia@chd.edu.cn (J.G.); 2025131023@chd.edu.cn (Q.L.); 2023904974@chd.edu.cn (Y.L.); 2025906098@chd.edu.cn (Y.Z.); 2 Saudi Arabian Oil Company, Dhahran 31311, Saudi Arabia; mohammed.mehthel@aramco.com

**Keywords:** asphalt VOCs, ZSM-5 zeolite, molecular simulation, adsorption mechanism, emission reduction

## Abstract

To reduce environmental pollution caused by volatile organic compounds (VOCs) released during asphalt application, various porous materials have been used to adsorb asphalt VOCs due to their rich pore structures. However, asphalt VOCs are so complex that emission reduction mechanisms still require further study. In this study, Materials Studio was used to simulate the molecular dynamics of asphalt VOC adsorption by ZSM-5 zeolite. The adsorption heat, capacity, and energy of ZSM-5’s adsorption of the main asphalt VOCs was obtained by means of molecular simulation to reveal the adsorption rules and selectivity. Zeolite model simulations with different structures were run to investigate possibilities for the optimization of ZSM-5. In addition, the actual VOC emission reduction effects of ZSM-5 in asphalt were compared with the MS simulation results. The VOC emission reduction mechanism was discussed based on both microscopic simulations and macroscopic verification. The results show that hydrocarbon derivative VOCs are more likely to be adsorbed due to their higher polarity. The smaller molecules of these VOCs are easier to adsorb because they occupy a smaller pore volume. When several molecules are mixed, competitive adsorption occurs. The selective adsorption probabilities of n-hexane, 1-methylcyclopentene, and toluene increase. In relation to the structure of zeolites, the Si/Al ratio and pore size of zeolites can both affect adsorption ability. A low Si/Al ratio can increase the number of surface acid active sites, while a micro–mesoporous structure increases the pore volume. The actual emission reduction data confirm that computational simulation has high accuracy in evaluating VOC emission reduction based on physical adsorption. Low-Si/Al-ratio and micro–mesoporous zeolites show better emission reduction ability for non-benzene VOCs than high-Si/Al-ratio and microporous zeolites. The emission reduction efficiency is up to 44%. However, the aromatization reaction was more easily catalyzed by zeolites, leading to the discrepancy between the simulated adsorption data and the actual situation. In future work, the boundary conditions and parameter settings of the simulations should be changed to achieve greater accuracy.

## 1. Introduction

The low-carbon and environmentally friendly development of road engineering materials has become an important direction in asphalt pavement construction [1,2]. During mixing, transport, paving, and the early service stage, asphalt materials may release volatile organic compounds (VOCs) under high temperatures [2,3]. This can cause material loss and air pollution [4,5]. It may also create risks to workers’ health and the surrounding environment [5,6]. Therefore, reducing VOC emissions from asphalt has become a key problem in green road construction [7].

At present, porous adsorbent materials have received widespread attention in material-based methods for reducing asphalt VOCs [7,8]. These porous adsorbents usually possess well-defined pore architectures, large specific surface areas, and tunable pore-size distributions. For zeolite adsorbents, the pore size, accessible pore volume, channel topology, and surface adsorption sites jointly determine the capture and diffusion behavior of VOC molecules [9]. They also have large specific surface areas and stable adsorption performance [10]. They mainly use their internal pores to physically adsorb asphalt VOC molecules [9]. VOC molecules can be captured and fixed through pore size matching, pore volume distribution, and surface adsorption sites [11]. This can reduce the release concentration of VOCs [12]. Compared with chemical reaction methods, physical adsorption usually works under mild conditions [13]. It also has better material adaptability and lower environmental risk [13]. International studies have also contributed to the understanding of asphalt fume emissions and zeolite-based VOC control. Thives reviewed the emissions and energy consumption of asphalt mixtures and highlighted the environmental benefits of warm-mix and recycled asphalt technologies [14]. These studies provide useful references for asphalt-emission mitigation and zeolite-based VOC control. However, most previous works focused on macroscopic emission characterization, single VOC molecules, or general VOC treatment systems, while the competitive adsorption of representative asphalt VOC mixtures on structurally regulated ZSM-5 zeolites remains insufficiently understood.

Regarding the microscopic mechanism, the physical adsorption of VOCs by porous materials mainly depends on intermolecular forces [13]. These forces include van der Waals forces, electrostatic interactions, and interactions between pore walls and adsorbate molecules [11]. When asphalt is heated, VOC molecules move and migrate [3]. Their diffusion, collision, adsorption, and desorption in the pores show clear molecular dynamics characteristics [11]. Therefore, molecular simulation can be used to study the adsorption behavior of asphalt VOCs in porous materials [15], revealing the emission reduction mechanism at the molecular scale. It can also make up for the limits of macroscopic experiments, which cannot directly observe microscopic processes [16].

In recent years, molecular simulation software such as Materials Studio (MS) has been widely used in studies of adsorption, diffusion, interface interaction, and material structure optimization [16]. By building models of adsorbate molecules and porous materials, researchers can calculate and analyze adsorption energy, adsorption configuration, diffusion behavior, and intermolecular interactions [16,17]. These results can provide a theoretical basis for evaluating adsorption performance and optimizing pore structure design [11]. Previous studies have shown that molecular simulation can help explain the adsorption rules of porous materials for gaseous pollutants or small organic molecules [9,16]. It can also provide microscopic support for the screening and modification of functional materials [12].

In this study, ZSM-5 zeolite was selected as the porous adsorbent because of its MFI-type microporous channel structure and molecular-sieving effect [16]. The adsorption of VOC molecules by ZSM-5 is closely related to channel accessibility, pore-size matching, pore volume, and surface adsorption sites rather than to an undefined “rich pore structure” [9]. Its adsorption of VOC molecules mainly occurs through physical adsorption [10], also called van der Waals adsorption [18]. At the microscopic level, it refers to the intermolecular forces between the adsorbate and the adsorbent [13]. These forces include dispersion forces, electrostatic forces, and induction forces [19], and are closely related to the force field of the adsorption surface [11,20].

Therefore, this study uses Materials Studio 2020 molecular simulation to explore the adsorption behavior and rules of ZSM-5 zeolite for single asphalt VOC molecules [21,22]. It also simulates the adsorption differences under mixed atmospheres of similar VOC molecules [23]. These simulations helped clarify the competitive adsorption behavior among VOC molecules and reveal the selective adsorption mechanism of ZSM-5 zeolite. Based on the main pollutant molecules in asphalt VOCs, a zeolite adsorption model was established from the perspective of microscopic simulation, and the selective design direction of zeolite materials was clarified. Furthermore, the accuracy of the simulation results was verified through actual emission reduction experiments, thereby establishing a closed loop of prediction and verification. Overall, this work provides new ideas, theoretical support, and data evidence for the structural optimization and efficient adsorption design of ZSM-5 zeolite-based asphalt VOC emission reduction materials [24].

## 2. Materials and Methods

### 2.1. Establishment of Zeolite and VOC Molecular Models

Three ZSM-5 zeolite models were built in this study. These were pure-silica microporous ZSM-5 zeolite (S-Mi), silicon-aluminum microporous ZSM-5 zeolite (SA-Mi), and silicon-aluminum micro–mesoporous ZSM-5 zeolite (SA-MiMe).

By comparing S-Mi and SA-Mi, the effect of Al atoms on the adsorption of asphalt VOCs can be analyzed. This can further help infer the influence of different Si/Al ratios on VOC emission reduction. By comparing the adsorption differences between SA-Mi and SA-MiMe, the adsorption rules and mechanisms of asphalt VOCs in zeolites with different pore structures can be studied.

**Figure 1:**
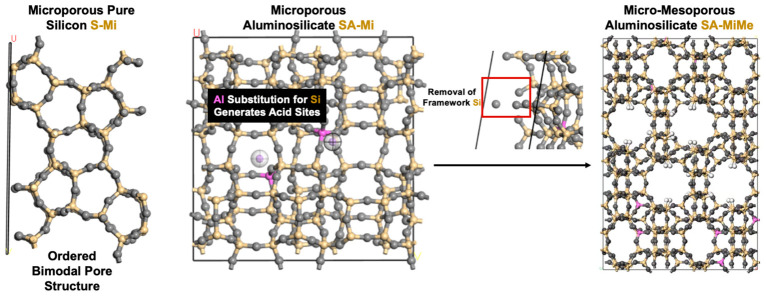
Models of three ZSM-5 zeolites: S-Mi, SA-Mi, and SA-MiMe.

The classic ZSM-5 zeolite model was obtained from the Database of Zeolite Structures. Its basic structural unit is the silicon-oxygen tetrahedron. The model contains 96 Si atoms and 192 O atoms. Some Si atoms were manually replaced by Al atoms. Specifically, two Si atoms in one unit cell were replaced by two Al atoms, giving an approximate Si/Al ratio of 50. When a silicon atom (Si^4+^) was substituted by an aluminum atom (Al^3+^), one proton (H^+^) was introduced onto a neighboring oxygen atom to maintain charge balance, thereby generating acid sites. Brønsted acid sites were formed inside the zeolite channels, while Lewis acid sites appeared on the pore surface. Some Si atoms were also removed to construct the micro–mesoporous structure. In each unit cell, four Si atoms adjacent to one selected Si atom, together with their connected O atoms, were removed, leading to the transformation from a microporous structure to a mesoporous structure. Finally, all unit cells were expanded into a 2 × 2 × 2 supercell to form a larger structural model for the subsequent adsorption simulations. The ball-and-stick models are shown in Figure 1.

Since the SA-Mi and SA-MiMe models were built from the pure-silica microporous zeolite model by atom substitution and manual desilication, their structures did not meet the minimum energy principle. Therefore, geometry optimization was carried out. After optimization, new zeolite structures and related data were obtained. Table 1 shows the lattice parameters of the three zeolite models after geometry optimization.

Thirteen main components of asphalt VOCs were selected as representative molecules for adsorption simulation. These molecules included four alkanes: n-hexane, n-heptane, 2-methylhexane, and 3-methylhexane. They also included four olefins: 1-hexene, 1-heptene, 1-methylcyclopentene, and 2-methyl-1-pentene. In addition, two hydrocarbon derivatives were selected: acetone and n-butyraldehyde. Two benzene series compounds were also selected: toluene and o-xylene. One polycyclic aromatic hydrocarbon (PAH), 3,6-dimethylphenanthrene, was also included.

**Figure 2:**
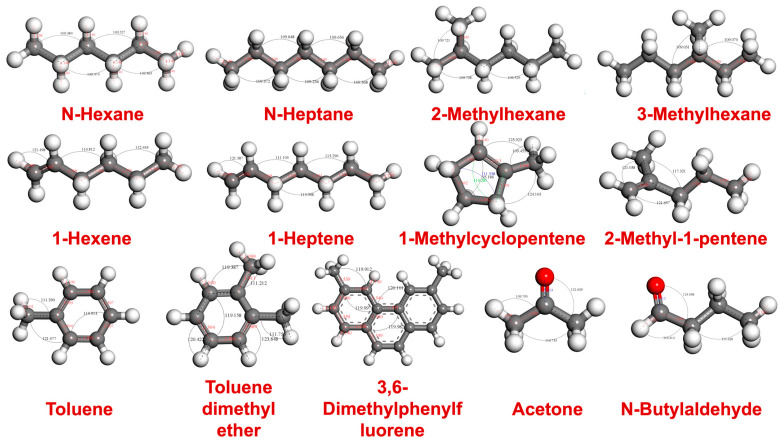
Molecular structures and bond angles of the 13 representative asphalt VOCs.

The molecular models of the 13 VOCs were built in Materials Studio (MS). Their ball-and-stick models, bond lengths, and bond angle parameters are shown in Figure 2. Each molecule was then optimized. After charges were assigned, the molecules were also placed in the COMPASS III force field. Commonly used force fields include COMPASS, CVFF, and PCFF. Among them, the COMPASS force field is a widely used molecular dynamics force field. It has a broad range of applicability and is suitable for inorganic materials, organic materials, metals, and their hybrid systems. Moreover, it can accurately describe various types of chemical bonds and intermolecular interactions. Compared with the original COMPASS force field, COMPASS III has been improved in several aspects. More importantly, it supports the Si and Al atoms in ZSM-5 zeolite structures, making it suitable for the present study.

**Figure 3:**
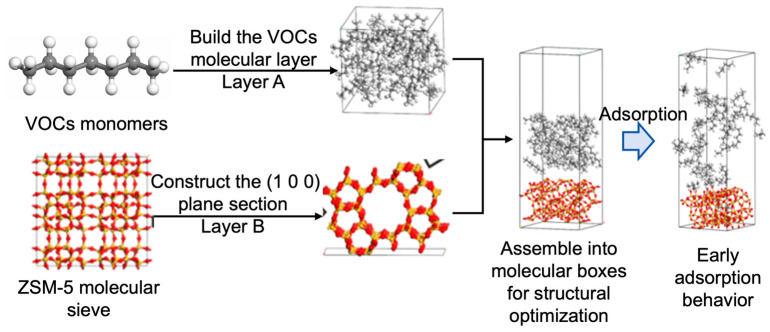
Flow chart of adsorption system construction.

Based on the models of the zeolite adsorbent and VOC adsorbates, the adsorption system was built according to the steps shown in Figure 3. The adsorption simulation was performed using the GCMC method. The Adsorption Isotherm and Fixed Pressure task modules in Sorption were employed, with the Metropolis algorithm adopted. The simulation temperature was set to 433 K, the number of equilibration steps was 1 × 10^4^, and the number of production steps was 1 × 10^5^. The simulated pressure range was 1–1000 kPa. For the molecular dynamics simulation, the Dynamics module in Forcite was used. The NVT ensemble and Berendsen thermostat were adopted, with the accuracy set to Medium. The simulation duration was 1000 ps, and the time step was 1000 fs.

Taking the adsorption of a single VOC as an example, a ball-and-stick model of one VOC molecule was first built. Then, the Amorphous Cell operation was performed to obtain an aggregate containing many single molecules. This aggregate was used to build the VOC molecular layer, named Layer A.

Next, the optimized ZSM-5 zeolite model was cut along the (1 0 0) crystal plane. For ZSM-5 zeolite, the (1 0 0) crystal plane corresponds to the pore openings of the channel system. Therefore, selecting this crystal plane enables an effective investigation of the relationship between the pore channels and VOC adsorption. In this way, an adsorbent zeolite layer with an exposed surface in contact with the VOC molecules was obtained. This layer was named Layer B [25].

Layer A and Layer B were then assembled into a new molecular box. To avoid the effect of periodicity, a 20 Å vacuum layer was added above the system [25]. After that, structural optimization was carried out. Following the minimum energy principle, the adsorption system was obtained. Then, the subsequent simulation of adsorption behavior was performed.

The adsorption simulation data in MS include saturated adsorption capacity, adsorption heat, and adsorption energy. All of these can be used to characterize adsorption performance. Adsorption capacity reflects the ability of the zeolite to adsorb VOCs.

Inside the pores, VOC molecules first undergo reversible Langmuir monolayer adsorption in the micropores. This process follows the model equation shown in Equation (1). The VOC molecules fill the micropore volume [26]. The turning point of the adsorption curve indicates the monolayer adsorption saturation of the micropores in the adsorbent.

(1) $$Q_{e}=KQ_{max}C_{e}/(1+C_{e})$$

where

$Q_{e}$ is the adsorption capacity per unit mass, mmol/g;

$Q_{max}$ is the maximum adsorption capacity, mmol/g;

$C_{e}$ is the equilibrium concentration;

K is the equilibrium constant.

The adsorption heat refers to the energy released when VOC molecules come into contact with the adsorbent surface. This is caused by the decrease in molecular velocity. It mainly reflects the heat produced during the dynamic process of VOC molecules. It can be used to evaluate the strength of molecular adsorption. A higher adsorption heat means that the molecule is more likely to be adsorbed on this surface.

Adsorption energy refers to the decrease in the total free energy of the system during adsorption. In this process, the adsorbate, namely VOCs, is adsorbed by the adsorbent, namely ZSM-5 zeolite. Adsorption energy reflects the interaction strength between the adsorbate and the adsorbent. A higher adsorption energy means a stronger interaction. It also means that ZSM-5 zeolite has a stronger adsorption ability for this VOC molecule. The system is more stable, and more energy is needed for desorption. The interaction energy is calculated by Equation (2).

(2) $$∆E=E_{total}-(E_{ZSM-5}+E_{VOC})$$

where

∆E—ZSM-5 is the interaction energy between ZSM-5 zeolite and the VOC molecule, kcal/mol;

$E_{total}$ is the total energy of the system, kcal/mol;

$E_{ZSM-5}$ is the energy of the ZSM-5 adsorbent, kcal/mol;

$E_{VOC}$ is the energy of the VOC adsorbate molecule, kcal/mol.

In MS simulation, adsorption is a continuous process. The computer can output energy data at regular time intervals. In this study, a script was written in Perl to calculate the adsorption energy at each step within 1000 ps. The adsorption energy of each step was then summarized into a scatter plot related to the simulation steps. This plot directly shows the energy release during the adsorption simulation of VOC molecules by the zeolite.

### 2.2. Synthesis and Emission Reduction Verification of Micro–Mesoporous ZSM-5 Zeolite

The raw materials used in this experiment included asphalt raw material and zeolite raw material. The asphalt was 70# base asphalt, whose basic properties are shown as Table 2.

The zeolite was commercial microporous ZSM-5 zeolite produced by the Catalyst Plant of Nankai University. Alkali treatment is a common method for preparing micro–mesoporous zeolites. In this experiment, NaOH alkali treatment was used to destroy Si atoms in the zeolite framework. In this way, random micro–mesoporous structures were formed. The VOC emission reduction effects of the original zeolite and the treated zeolite were then compared, whose properties are shown as Table 3. This was used to verify the simulation results from MS.

The zeolite was used as an additive. It was added into asphalt at a content of 2% to prepare modified asphalt. Firstly, 100 g of asphalt was heated from room temperature to 160 °C and kept at this temperature for 1 min. Then, VOCs were collected for 30 s at a flow rate of 0.1 L/min using a CNW T-C stainless steel thermal desorption tube. The collected adsorption tubes were tested by means of thermal desorption–gas chromatography–mass spectrometry (TD-GC-MS). The instrument model was Agilent 7890B-5977B from Agilent Technologies, Santa Clara, CA, USA.

### 2.3. Research Technical Route

**Figure 4:**
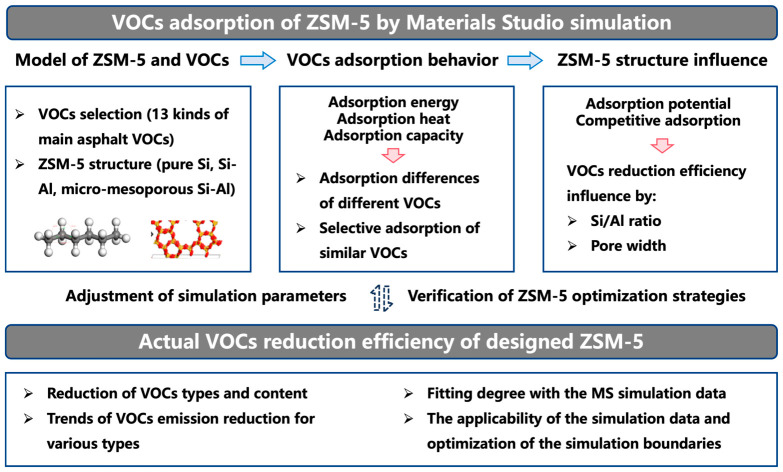
Research technical route.

This study investigated the adsorption of asphalt VOC molecules by zeolites. Materials Studio (MS) was used to calculate the interactions between atoms and molecules. The simulation results were then verified by the actual emission reduction effect, as shown in Figure 4.

## 3. Results and Analysis

### 3.1. Adsorption Mechanism of Asphalt VOCs on ZSM-5

The porous structure of ZSM-5 can promote the adsorption of VOC molecules. In this study, molecular dynamics simulation was carried out using Materials Studio. The aim was to clarify the adsorption mechanism and adsorption selectivity of VOC molecules in the pores.

#### 3.1.1. Adsorption Behavior of Single Molecules

ZSM-5 zeolite contains a microporous channel system, and the adsorption of asphalt VOC molecules is controlled by the matching relationship between molecular size and pore dimensions, as well as by the interaction between VOC molecules and pore-wall adsorption sites. The adsorption of asphalt VOC molecules on ZSM-5 mainly occurs through physical adsorption. By building adsorption boxes, the adsorption simulation of 13 asphalt VOC molecules on ZSM-5 zeolite was completed. The adsorption capacity, adsorption energy, and adsorption heat were obtained. The adsorption behavior of single molecules was also studied.

##### Saturated Adsorption Capacity

**Figure 5:**
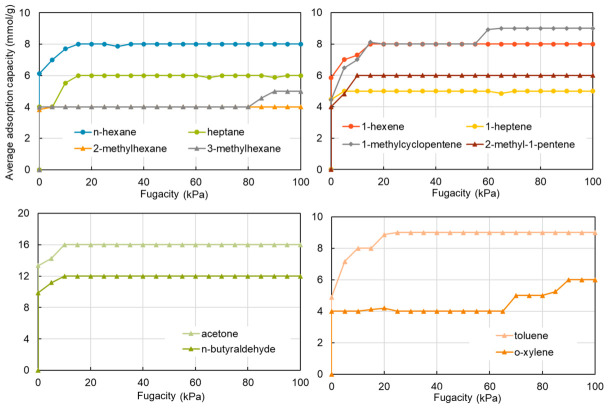
Saturation adsorption curves of 12 VOC molecules by ZSM-5.

Figure 5 shows the saturated adsorption capacity curves of 12 VOC molecules on ZSM-5. Among them, since the molecular size of 3,6-dimethylphenanthrene is too large to enter the pores, the adsorption capacity cannot reach the simulated value. These curves can be fitted as adsorption isotherms of VOCs on the zeolite. Under high fugacity, the stable value of adsorption capacity can be regarded as the saturated adsorption capacity of the zeolite for each VOC.

The adsorption capacity curves of different molecules show different shapes. However, they all show a similar trend. When the fugacity is between 0 and 10 kPa, the average adsorption capacity increases rapidly. Then, the adsorption reaches the monolayer adsorption saturation state in micropores. The monolayer saturated adsorption capacity is different for different VOC molecules.

As the fugacity increases, the adsorption capacity of some VOCs increases again after the first platform stage. These VOCs include 3-methylhexane, 1-methylcyclopentene, toluene, and o-xylene. This indicates that multilayer adsorption occurs in the pores for these four substances. Among these four VOCs, three contain benzene rings or cyclic structures and one contains a branched chain. Therefore, the pore superposition effect causes multilayer adsorption of VOC molecules. Finally, with the continuous increase in fugacity, the adsorption of each VOC reaches its maximum adsorption capacity. This value is the adsorption limit of the zeolite for that VOC.

The adsorption capacity of VOCs in the same category was compared. The results show that VOCs with smaller molecular weights usually have higher average adsorption capacities than VOCs with larger molecular weights in the same category. Toluene and o-xylene are both benzene series compounds. The average adsorption capacity of toluene is 9 mmol/g, which is 1.5 times that of o-xylene. Therefore, molecular weight has a large influence. Because the pore volume of the zeolite is limited, the maximum adsorption capacity for small molecules is usually slightly higher than that for large molecules.

Among alkanes, 2-methylhexane and 3-methylhexane show similar adsorption trends and adsorption capacities at low fugacity, whereas their isomer n-heptane exhibits a higher adsorption capacity. A similar phenomenon is also observed for olefin isomers, where the adsorption capacity of 1-hexene is higher than that of 2-methyl-1-pentene. It is therefore inferred that, for hydrocarbon VOC isomers, the presence of branched chains may hinder molecular entry into the zeolite channels, resulting in relatively lower adsorption capacity.

In addition, among different types of VOCs, hydrocarbon derivatives have an adsorption capacity as high as 16 mmol/g. This value is clearly higher than that of other molecules. This is because aldehydes and ketones have stronger polarity. Due to the combined effect of polarity, they are more likely to be adsorbed on the zeolite surface and inside the pores. Therefore, their adsorption capacity is higher.

##### Adsorption Heat

**Figure 6:**
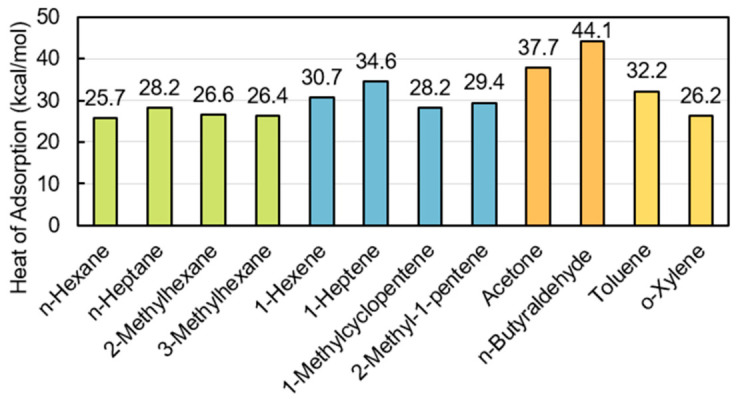
Adsorption heat of 12 VOC molecules by ZSM-5.

Figure 6 compares the simulated adsorption heat of 12 VOC molecules. Similarly, the simulated adsorption heat of 3,6-dimethylphenanthrene cannot be reached due to its large molecular size. The adsorption heat of physical adsorption ranges from 5 to 40 kJ/mol, while chemical adsorption ranges from 80 to 400 kJ/mol. The adsorption heat values in Figure 6 range from 20 to 45 kcal/mol (80–180 kJ/mol), indicating that the adsorption of VOCs by the zeolite includes both physical adsorption and chemical adsorption.

Among these VOCs, acetone and n-butyraldehyde show higher adsorption heat than other molecules. This is because they contain highly active -C=O- and -CHO groups, which are more easily activated by acid sites on the zeolite surface. The -CHO group is readily protonated at acid sites, followed by reactions such as nucleophilic addition and condensation. This is because the carbonyl oxygen contains lone-pair electrons, while the carbonyl carbon bears a strong δ+ charge, enabling the formation of strong hydrogen-bonding and electrostatic interactions with Si–OH–Al sites in the ZSM-5 framework. Ketone groups (-C=O) can also form hydrogen bonds; however, because the positive charge on the carbonyl carbon is partially dispersed by alkyl groups, their adsorption strength is generally weaker than that of aldehyde groups but stronger than that of -CH_3_ groups. Under the combined effects of steric hindrance and electron-cloud density, the reactivity follows the order -CHO > -C=O >> -CH_3_. As a result, partial chemical reactions may occur during adsorption, leading to higher adsorption heat than that of other molecules.

##### Adsorption Energy

**Figure 7:**
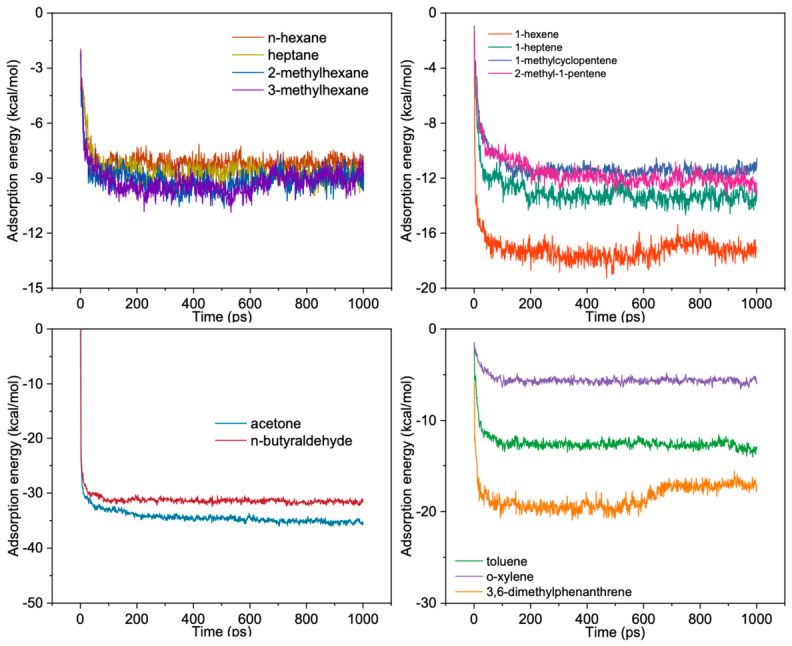
Adsorption energy change of 13 VOC molecules by ZSM-5.

Figure 7 summarizes the adsorption energy curves of 13 asphalt VOC molecules on micro–mesoporous ZSM-5 zeolite. The data show that the adsorption fitting of VOCs by the zeolite changes greatly within the first 100 ps. The adsorption energy basically reaches equilibrium between 100 and 200 ps. The adsorption simulation process is almost completed within the first 200 ps. After that, the adsorption energy fluctuates around a stable value.

VOCs of the same type were then compared. The results show that the adsorption energy differences among the four alkane molecules are small. Their adsorption energies overlap in the range of 8–10 kcal/mol.

For olefins, except for 1-hexene, the adsorption energies of the other three olefin molecules show little difference. However, the adsorption energy of 1-hexene is much higher than those of the other three olefins. ZSM-5 has excellent adsorption ability for 1-hexene. This may be because 1-hexene has a smaller cross-sectional size. Therefore, it can enter the zeolite pores more easily and be adsorbed.

The adsorption energies of hydrocarbon derivatives and benzene series compounds show large differences. This is mainly because they are strongly affected by molecular polarity and structural size. The adsorption trend shows that small molecules have higher adsorption energy than molecules with larger molecular weights. This is consistent with the trend of adsorption capacity. Small molecules have smaller molecular sizes, and so they can enter the pores more easily. Due to the pore effect, they are more difficult to desorb. Therefore, they show higher adsorption energy.

**Figure 8:**
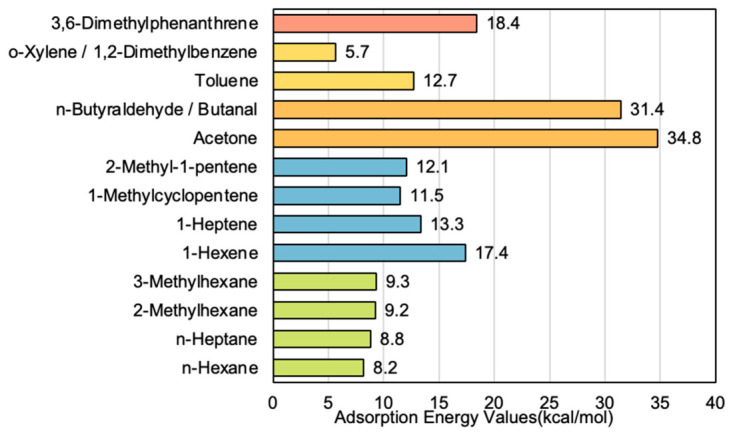
Adsorption energy of 13 VOC molecules by ZSM-5.

Since the adsorption of each VOC molecule by the zeolite reached equilibrium after 200 ps, the average adsorption energy from 200 to 1000 ps was selected as the adsorption energy of the zeolite for each VOC. The results are summarized in Figure 8.

The data show that the adsorption energies of n-butyraldehyde and acetone are much higher than those of the other 11 VOCs. Their adsorption energies are 31.4 kcal/mol and 34.8 kcal/mol, respectively. This is because hydrocarbon derivatives contain oxygen-containing groups. These groups give them high polarity of about 4.5. Therefore, two highly polar molecules are more easily adsorbed by ZSM-5.

The adsorption energy of olefins is slightly higher than that of alkanes. This is because the double bonds in olefins make their polarity slightly higher than that of alkanes. As a result, olefins are more easily adsorbed by ZSM-5.

For toluene and o-xylene, the adsorption energy of the smaller molecule, toluene, is clearly higher than that of o-xylene. Due to polarity, the adsorption energy of toluene is higher than that of most alkanes and olefins. However, the adsorption energy of o-xylene is lower than that of all alkanes and olefins. This is mainly because o-xylene has a larger molecular size. Although its polarity is much higher than that of alkanes and olefins, its benzene ring structure and methyl groups make its molecular size slightly larger than the micropore size. Therefore, it is limited by the pore opening. It is more difficult for o-xylene to enter the internal pores of the zeolite. It is mainly adsorbed on the zeolite surface. This leads to a lower adsorption energy.

The molecular size of 3,6-dimethylphenanthrene is even larger. Therefore, it cannot easily enter the pores. Its adsorption capacity curve is 0. However, it has π bonds formed by three benzene rings. Compared with other alkane and olefin VOC molecules, it has stronger interactions with the zeolite surface. Therefore, its adsorption energy is slightly higher than that of other weakly polar molecules.

#### 3.1.2. Adsorption Selectivity of Similar VOCs

**Figure 9:**
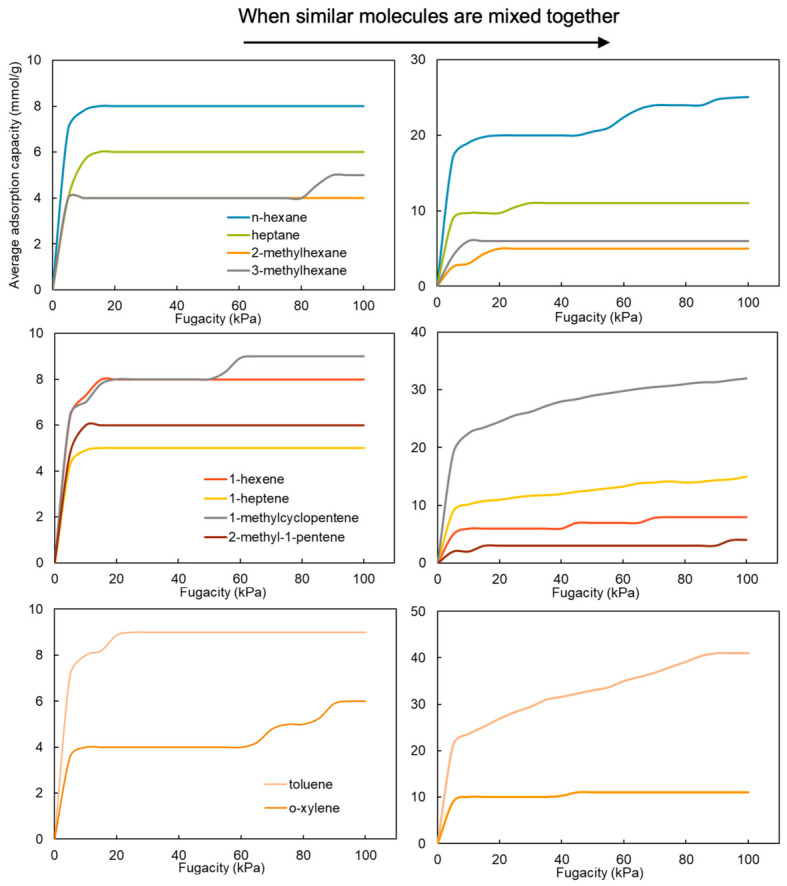
Simulated adsorption isotherm of ZSM-5 in mixed gas atmosphere.

Adsorption simulations were carried out for similar VOC molecules with ZSM-5. The simulated systems included four alkanes, four olefins, and two benzene series compounds. After the simulations, adsorption capacity curves of 10 VOCs were obtained. The data points were smoothed and fitted. The adsorption isotherms of VOC molecules are shown in Figure 9.

The adsorption isotherms in the mixed atmosphere are quite different from those in the single-molecule atmosphere. For all types of VOCs, the maximum adsorption capacity increases compared with single-molecule adsorption. The minimum adsorption capacity decreases. The difference in adsorption capacity among different VOC molecules becomes larger. The simulations also confirm that competitive adsorption occurs among similar VOC molecules in the mixed atmosphere. This competitive adsorption behavior can be further explained by pore confinement and intermolecular interactions. In the confined microporous channels of ZSM-5, the available pore volume and adsorption sites are limited. Therefore, VOC molecules with smaller molecular size or better pore-size matching can preferentially enter the channels and occupy adsorption sites. Once these molecules are adsorbed, they may hinder the diffusion and adsorption of other molecules through spatial occupation. In addition, adsorbed VOC molecules can interact with neighboring molecules through van der Waals interactions and induced dipole interactions, which further affects the stability of the adsorbed phase. As a result, the adsorption capacity of some VOCs increases in the mixed atmosphere, whereas that of other molecules decreases, leading to selective adsorption.

For alkane VOCs, the data show that only the saturated adsorption capacity of n-hexane increases among the four alkanes. The saturated adsorption capacities of the other three alkanes decrease. This indicates that when these four VOC molecules exist at the same time, ZSM-5 adsorbs more n-hexane at the cost of reducing the adsorption of other alkane molecules. This may be because n-hexane has the smallest molecular size. In the mixed atmosphere, it first undergoes monolayer adsorption in the pores. Then, multilayer adsorption occurs. This further increases its adsorption capacity. The adsorption capacity of n-heptane slightly decreases in the mixed atmosphere. The two branched isomers have similar adsorption capacities in single-molecule adsorption. Their adsorption capacities also decrease at the same time in the mixed atmosphere.

The adsorption data of olefins show that the saturated adsorption capacities of 1-methylcyclopentene and 1-heptene increase. However, the saturated adsorption capacities of 1-hexene and 2-methyl-1-pentene decrease greatly. ZSM-5 preferentially adsorbs 1-methylcyclopentene and 1-heptene. The ring structure of 1-methylcyclopentene may make it easier to confine in the pores after it enters them. This is caused by steric hindrance. As a result, its desorption probability decreases. For 1-heptene, its molecular weight and molecular size are larger. It may therefore be adsorbed due to the pore superposition effect.

The adsorption data of the two benzene series compounds show that the adsorption capacity still follows the order of toluene > o-xylene. In the mixed atmosphere, the difference between their adsorption capacities becomes larger. The adsorption curve of toluene still shows an increasing trend when the fugacity reaches 100 kPa. This is similar to 1-methylcyclopentene. At this point, toluene has not yet reached adsorption saturation. When the pressure continues to increase, its adsorption capacity may still increase. Toluene has strong adsorption ability.

**Figure 10:**
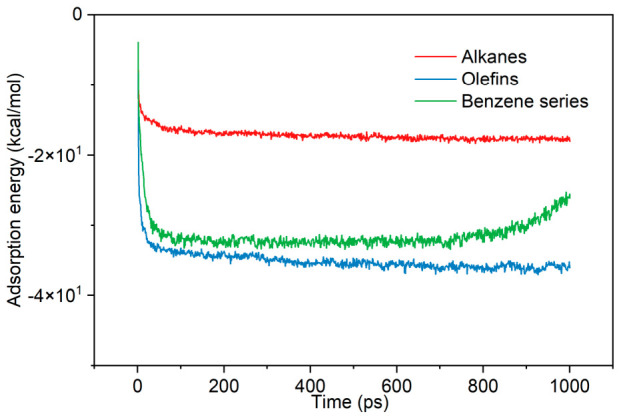
Adsorption energy change of three types of VOCs by ZSM-5.

The adsorption heat only reflects the state change of the adsorbate during adsorption. It is not related to the adsorption atmosphere. However, adsorption energy reflects the energy change of the adsorption system. It is closely related to the adsorption atmosphere. Figure 10 shows the adsorption energy curves of alkanes, olefins, and benzene series compounds in the mixed atmosphere. Similar to single-molecule adsorption, the adsorption process basically reaches equilibrium within the first 200 ps. The data show that the adsorption ability follows the order of olefins > alkanes. ZSM-5 has stronger adsorption ability for olefins than for alkanes.

**Figure 11:**
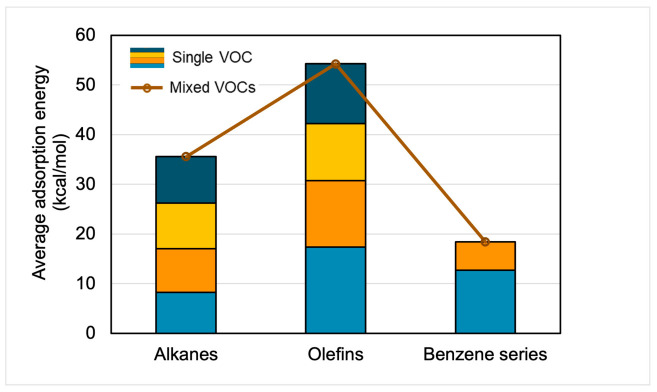
Adsorption energy changes in mixed gas atmosphere and single molecule atmosphere.

Figure 11 compares the average adsorption energy values in the mixed atmosphere and the single-molecule atmosphere. The data show that the average adsorption energies of alkanes and benzene series compounds are similar in the two atmospheres. The molecular atmosphere has little effect on the overall energy change of adsorption. However, the adsorption energy of olefins in the mixed atmosphere is clearly higher than that in the single-molecule atmosphere. The coexistence of several olefin molecules can promote the adsorption of the system. It is inferred that different olefins may promote each other when they exist at the same time. They may also enhance intermolecular interactions. This strengthens the confinement of molecules on the zeolite surface and inside the pores. Therefore, the overall adsorption effect is improved.

In summary, in the adsorption simulation, adsorption heat mainly describes the adsorption process of VOCs. It is only related to the adsorbate and the adsorbent. It is not affected by the VOC atmosphere. However, adsorption capacity and adsorption energy are strongly affected by the VOC atmosphere. In the mixed atmosphere, competitive adsorption occurs among VOCs. This finally leads to selective adsorption.

In the atmosphere of similar VOCs, ZSM-5 shows strong selective adsorption for n-hexane, 1-methylcyclopentene, and toluene. This may be caused by the effects of molecular size and polarity.

### 3.2. Effect of Zeolite Structure on Adsorption

Previous results showed that the adsorption of VOC molecules on zeolites comprises both physical adsorption and chemical adsorption. Physical adsorption mainly depends on the pore structure and surface roughness of the zeolite. Chemical adsorption depends on the chemical groups on the inner pore walls and the catalytic reactions at acid active sites. Therefore, the structural properties of zeolites have a strong effect on the adsorption performance and adsorption rules of VOCs.

#### 3.2.1. Si/Al Ratio

The S-Mi zeolite is composed of pure Si. It only reflects the pore intersection structure of ZSM-5 zeolite. The SA-Mi zeolite is built by replacing some Si atoms with Al atoms in the pure Si framework. Its Si/Al ratio is 50. The introduction of Al can attract positive charges and form acid active sites. It can also produce B acid sites on the surface and L acid sites in the pores. These acid sites can affect the adsorption of VOCs to some extent.

##### Effect on Adsorption Ability

**Figure 12:**
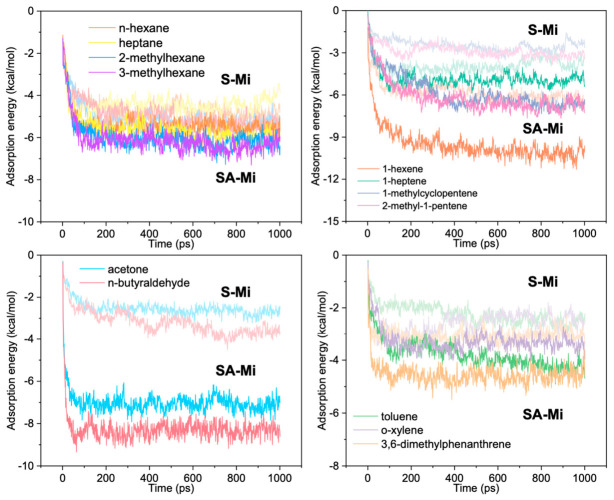
Effect of Si/Al ratio on the adsorption energy of 13 VOC molecules.

Figure 12 compares the effect of the Si/Al ratio on the adsorption energy of 13 VOCs. Overall, the adsorption energies of the 13 molecules all follow the order of SA-Mi > S-Mi. Replacing Si atoms with Al atoms can improve the surface acidity. It can also improve the adsorption ability of the zeolite for different VOCs.

The change in the Si/Al ratio has little effect on the adsorption ability for olefin VOCs. The adsorption energies of 1-heptene on SA-Mi and S-Mi are close. The introduction of Al atoms cannot greatly improve the adsorption ability for 1-heptene. However, it has different effects on the adsorption of the other three olefins.

For the two hydrocarbon derivatives, the adsorption energy differences between the two zeolites are small. Their adsorption energy curves almost overlap. After Al atoms are introduced, the adsorption energy doubles. Al introduction can improve the adsorption ability for VOCs with higher polarity.

For benzene series VOCs, the adsorption energy increases after Al introduction. This shows that Al atoms can promote stronger adsorption.

**Figure 13:**
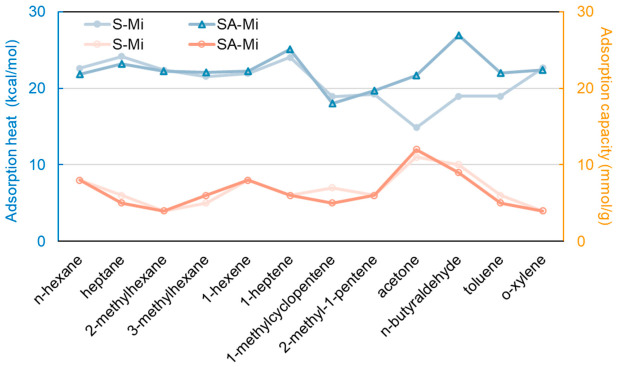
Effect of Si/Al ratio on the adsorption capacity and adsorption heat of voc molecules.

In addition to adsorption energy, the Si/Al ratio also affects the adsorption heat and adsorption capacity of VOCs. This reflects the influence of zeolite structure, as shown in Figure 13.

The adsorption heat data show that, except for acetone and n-butyraldehyde, the adsorption heat values of other VOCs on S-Mi and SA-Mi are basically the same. Compared with the pure-silica zeolite, the Al-containing zeolite increases the adsorption heat of acetone, n-butyraldehyde, and toluene. The adsorption heat of other molecules remains almost unchanged. This shows that the acid distribution on the zeolite surface can affect the adsorption of hydrocarbon derivatives and benzene series compounds.

This also indicates that the Si/Al ratio has a greater effect on the adsorption heat of polar molecules. A lower Si/Al ratio gives better adsorption ability for molecules with higher polarity. Compared with S-Mi, SA-Mi shows no obvious change in adsorption capacity. Therefore, the change in Si/Al ratio does not directly affect the adsorption capacity for VOC molecules. Its effect on adsorption capacity is not significant.

##### Effect on Competitive Adsorption

**Figure 14:**
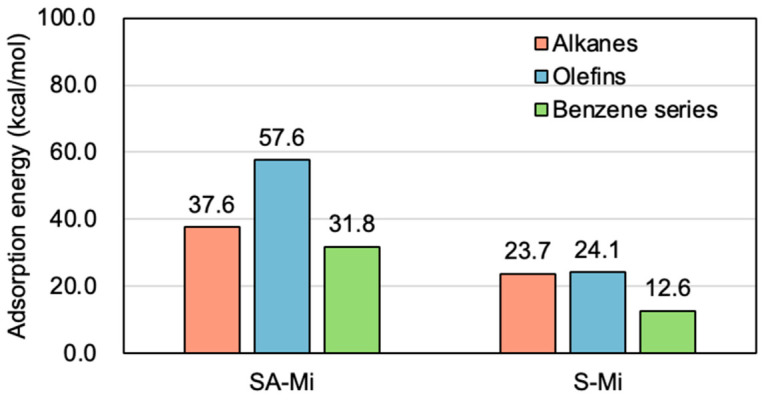
Effect of Si/Al ratio on the average adsorption energy of three types of VOCs.

Figure 14 compares the average adsorption energies of three types of VOCs, including alkanes, olefins, and benzene series compounds, on zeolites with different Si/Al ratios. The overall adsorption energy of SA-Mi is about two times higher than that of S-Mi. This is because the charge centers formed by Al atoms in the zeolite can enhance the intermolecular interactions with VOCs. As a result, the adsorption energy increases.

**Figure 15:**
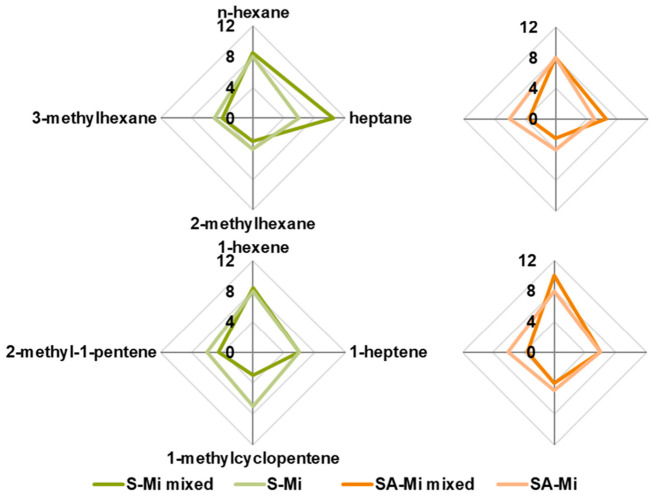
Effect of Si/Al ratio on the competitive adsorption of alkane and olefin VOC molecules.

The difference in adsorption capacity between the mixed atmosphere and the single-molecule atmosphere can reflect the effect of zeolite properties on competitive adsorption. Figure 15 compares the effect of different Si/Al ratios on the competitive adsorption of alkane and olefin VOC molecules.

The data show that, for the four alkane VOCs, S-Mi and SA-Mi show the same trend in competitive adsorption. Both zeolites promote the adsorption of n-heptane and reduce the adsorption of 3-methylhexane. The pure-silica microporous zeolite shows stronger selective adsorption for n-heptane.

For the four olefin VOCs, the two zeolites also show the same competitive adsorption trend. Both zeolites promote the adsorption of 1-hexene and reduce the adsorption of 1-methylcyclopentene and 2-methyl-1-pentene. The Al-containing zeolite shows stronger selective adsorption ability for 1-hexene.

Overall, the presence or absence of Al does not change the selective adsorption tendency for alkane and olefin VOCs. Both zeolites promote the selective adsorption of n-heptane and 1-hexene. However, the pure-Si zeolite has a stronger selective promotion effect for alkanes. The Al-containing zeolite has a stronger selective promotion effect for olefins.

#### 3.2.2. Pore Size

The SA-MiMe zeolite was prepared based on SA-Mi by means of structural desilication. This treatment caused some micropores to expand into mesopores. As a result, a zeolite structure with both micropores and mesopores was formed. The change in spatial structure also leads to differences in the simulated adsorption of VOC molecules.

##### Effect on Adsorption Ability

**Figure 16:**
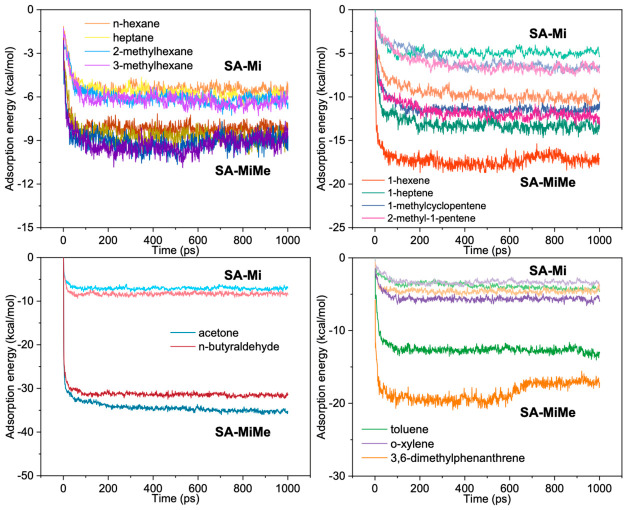
Effect of pore size on the adsorption energy of 13 VOC molecules.

Figure 16 compares the effect of pore size on the adsorption energy of 13 VOCs. In general, the adsorption energies of all 13 molecules follow the order of SA-MiMe > SA-Mi. The increase from SA-Mi to SA-MiMe is much larger than the increase from S-Mi to SA-Mi discussed in Section Effects on Adsorption Capacity. This shows that the introduction of mesopores can further improve VOC adsorption on the basis of improved surface acidity. As a result, the adsorption performance is greatly enhanced.

For 1-heptene, the adsorption energies on SA-Mi and S-Mi are similar. After the mesoporous structure is introduced, the adsorption energy increases to about 13.3 kcal/mol. This shows that the adsorption ability is greatly improved. The adsorption of 1-heptene is more affected by zeolite pore size than by acid site distribution.

In addition, compared with SA-Mi, the equilibrium adsorption energy of SA-MiMe is greatly improved. This is especially clear for hydrocarbon derivatives and benzene series compounds. The adsorption differences among similar molecules also become larger. The micro–mesoporous structure has a greater effect on the adsorption of highly polar molecules. It also shows selectivity. Molecules with smaller structures have higher adsorption potential.

**Figure 17:**
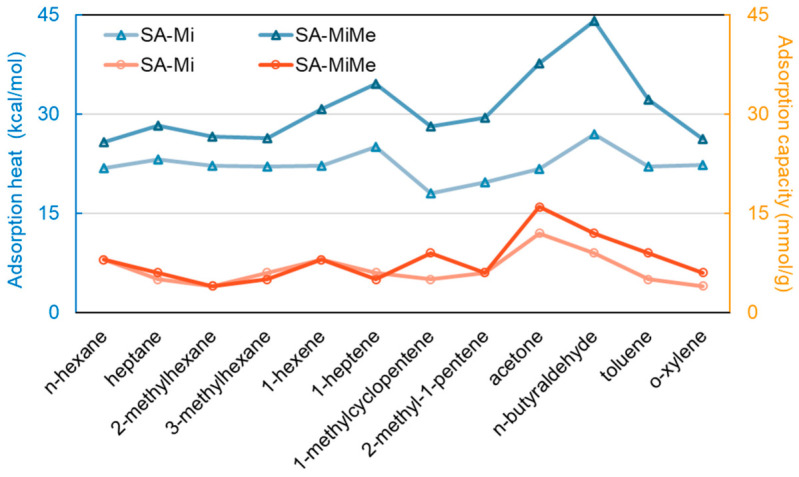
Effect of pore size on the adsorption capacity and adsorption heat of VOC molecules.

Figure 17 compares the effect of different pore sizes on the adsorption heat and adsorption capacity of VOCs. The data show that, based on the Al-containing zeolite, the micro–mesoporous zeolite greatly increases the adsorption heat of 12 VOCs. The increase follows the order of hydrocarbon derivatives > olefins > benzene series compounds > alkanes. This again proves that the change in zeolite structure has a stronger effect on the adsorption ability for polar VOCs.

For adsorption capacity, SA-MiMe introduces mesopores and increases pore volume. However, it does not clearly increase the adsorption capacity for alkanes and olefins. For the two hydrocarbon derivatives and the two benzene series compounds with higher polarity, SA-MiMe clearly increases the adsorption capacity. The adsorption capacity of VOC molecules is mainly related to the pore volume of the zeolite. After mesopores are introduced, the adsorption of highly polar molecules is effectively improved. At the same time, pore expansion increases the pore size. This increases the probability that large VOC molecules can enter the pores. Therefore, their adsorption ability is improved.

##### Effect on Competitive Adsorption

**Figure 18:**
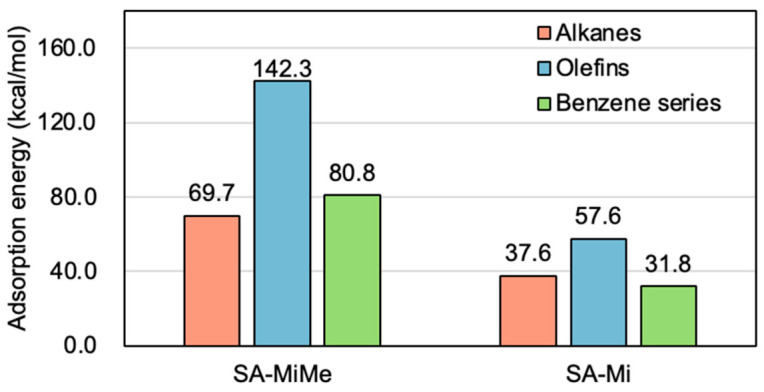
Effect of pore size on the average adsorption energy of three types of VOCs.

Figure 18 shows the effect of pore size on the average adsorption energy of three types of VOCs. The data show that when some micropores in the zeolite structure are expanded into mesopores, the adsorption energy of the three types of VOCs increases by about two times. The micro–mesoporous structure allows more large molecules to enter the pores. As a result, the overall adsorption ability for asphalt VOC molecules is effectively improved.

**Figure 19:**
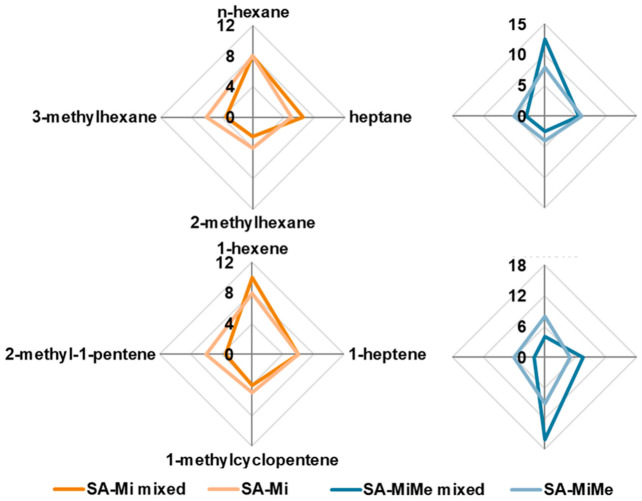
Effect of pore size on the competitive adsorption of alkane and olefin VOC molecules.

Figure 19 compares the competitive adsorption of similar VOCs under different pore sizes. The effects of SA-Mi and SA-MiMe on the competitive adsorption of alkane and olefin VOCs are quite different.

For the four alkane VOCs, n-heptane has stronger adsorption competitiveness in the microporous zeolite. However, n-hexane has stronger adsorption competitiveness in the micro–mesoporous zeolite.

For the four olefin VOCs, SA-Mi tends to adsorb 1-hexene. In contrast, SA-MiMe tends to adsorb 1-methylcyclopentene and 1-heptene. This is especially clear for 1-methylcyclopentene. The selective adsorption of this molecule by the zeolite is greatly improved. This may be because the micro–mesoporous structure creates larger pore space. After cyclic molecules such as 1-methylcyclopentene and large molecules such as 1-heptene enter the pores, they are affected by steric hindrance. They are not as easy to desorb as small molecules. Therefore, their adsorption capacity increases greatly. This also shows that the zeolite has a strong selective adsorption ability for them.

In summary, different zeolite structures have different effects on the simulated adsorption of VOC molecules. After Al is introduced into the zeolite, the distribution of surface acid active sites changes. This can improve the average adsorption energy of all VOC molecules. However, the increase is relatively small. The improvement in adsorption heat is mainly shown for hydrocarbon derivatives and benzene series compounds with higher polarity. The adsorption capacity does not change clearly.

After micro–mesopores are introduced, the pore volume of the zeolite increases. Therefore, the adsorption capacity of VOC molecules is improved. This is especially clear for benzene series compounds and hydrocarbon derivatives. In addition, the adsorption heat and adsorption energy of VOC molecules are greatly improved. The increase follows the order of hydrocarbon derivatives > benzene series compounds and olefins > alkanes. The micro–mesoporous structure strengthens the interaction between VOC molecules and the zeolite. It promotes adsorption on the surface and inside the pores. It also has a stronger improvement effect for substances with higher polarity.

### 3.3. Actual Emission Reduction Effect of Structural Regulation

To verify the relationship between actual emission reduction and simulated adsorption, Table 4 compares the actual emission reduction efficiency of the micro–mesoporous zeolite for 12 VOCs with the simulated adsorption data. The results confirm that the actual emission reduction trend for alkanes is basically consistent with the adsorption energy trend. The adsorption efficiencies for heptane and n-hexane are higher, while the emission reduction efficiencies of the other two molecules are slightly lower.

For olefins, the actual emission reduction efficiency also shows the same trend as the theoretical simulated adsorption heat. Both results show a very high emission reduction effect for 1-hexene.

For benzene series compounds, the simulated adsorption heat of toluene is higher than that of alkanes. In theory, this should lead to a good emission reduction effect for toluene. However, in the actual emission reduction test, both toluene and o-xylene show increased emissions. This is because adsorption is usually simulated by setting boundary conditions and adsorption parameters. However, in actual zeolite adsorption, the gas atmosphere is more complex. In addition, the catalytic effect of the zeolite may cause complex organic compounds to undergo chemical side reactions [27]. These processes cannot be simply realized in computer simulation. Therefore, for benzene series compounds, the actual emission reduction results are different from the simulation results.

The zeolite emission reduction data obtained by computational simulation are more suitable for VOC molecules mainly controlled by physical adsorption. Their adsorption energy can more accurately reflect the adsorption potential of the zeolite for different VOC molecules. The simulation can also explain the adsorption and emission reduction mechanism from the microscopic thermodynamic level.

However, for VOCs that may undergo catalytic reactions or other chemical reactions, an adsorption model based only on pore structure and surface electron distribution is no longer fully suitable. To simulate these processes, the boundary conditions and parameter settings need to be further modified. This can help meet more complex interaction conditions.

**Figure 20:**
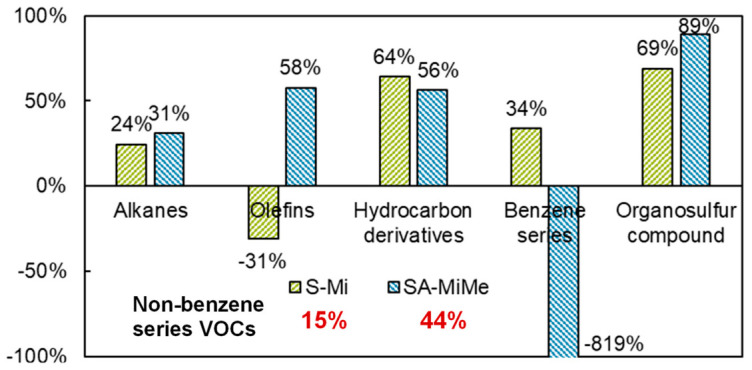
Comparison of actual VOC emission reduction of asphalt between S-Mi and SA-MiMe.

Figure 20 compares the actual emission reduction of asphalt VOCs between S-Mi and SA-MiMe zeolites. The data show that when the aluminum content increases and the pore size becomes larger, the reduction effects on alkanes, olefins, and organosulfur compounds are greatly improved. However, this also leads to an increase in benzene series compounds. This result is consistent with previous conclusions. Zeolites do not only rely on physical adsorption in asphalt emission reduction. Their catalytic effect also influences the chemical reactions of VOC molecules. When the pore size increases, it provides more space for chemical reactions. Brønsted acid sites inside the zeolite channels can regulate the catalytic process through the shape-selective effect of the pore structure, while Lewis acid sites on the pore surface may induce secondary reactions of the catalytic products. The presence of Brønsted and Lewis acid sites in the zeolite promotes the aromatization of VOCs within the channels. First, long-chain alkanes are cracked on Lewis acid sites on the zeolite surface to form highly active carbocations, producing small-molecule alkanes and olefins, such as ethylene and propylene. Subsequently, some small olefin molecules undergo polymerization at Brønsted acid sites to generate C6–C8 olefins. These olefin molecules then form aromatic precursors containing six-membered rings through exothermic reactions such as isomerization and cyclization. Finally, the aromatic precursors undergo exothermic dehydrogenation at Lewis acid active sites of the zeolite, resulting in the formation of benzene series compounds. This leads to the generation of more benzene series compounds. If the emission of benzene series compounds is not considered, the structurally optimized zeolite can increase the emission reduction efficiency from 15% to 44%. Therefore, the zeolite optimization approach obtained through MS calculations and simulations is proven feasible in the design of emission reduction materials that mainly rely on physical adsorption.

## 4. Conclusions

This study uses Materials Studio to simulate the adsorption of asphalt VOCs by zeolites at the microscopic level. It also uses actual experiments on VOC emissions from heated asphalt. Based on these, the study discusses the VOC reduction mechanism and zeolite structure design ideas. The following conclusions are drawn.

(1) The adsorption curves confirm that multilayer adsorption occurs inside the zeolite pores. The simulated adsorption data of hydrocarbon derivatives VOCs are higher than alkane, olefins and benzene series VOCs due to their high polarity. Small-molecule VOCs generally have higher average adsorption capacity than larger-molecule VOCs due to the limited pore volume of zeolites. Competitive adsorption will appear when there are complex VOCs. Zeolites have strong selective adsorption for n-hexane, 1-methylcyclopentene, and toluene, which is likely due to the influence of molecular size and polarity.
(2) The Si/Al ratio and pore size of zeolites have differential effects on the simulated adsorption of VOCs. When Si atoms are replaced by Al atoms, the distribution of surface acid active sites changes. This slightly increases the average adsorption energy. Micro–mesoporous zeolites, possessing a larger pore volume, significant increase the adsorption capacity. ZSM-5, which contains Al atoms and has a mesoporous structure, represents a target for optimization.
(3) Actual emission reduction data confirm that computational simulation can evaluate the reduction ability for non-benzene VOCs from a physical perspective. The low-Si/Al-ratio and micro–mesoporous zeolite achieves a reduction efficiency of 44% for non-benzene VOCs. However, the catalytic effect of zeolites leads to aromatization between benzene series VOCs. As a result, the simulation does not match reality, and thus further setting of simulation boundaries and parameters is needed to improve fitting accuracy when benzene series VOCs are simulated.

## Figures and Tables

**Table 1:** Molecular lattice parameters of three ZSM-5.

Parameter		S-Mi	SA-Mi	SA-MiMe
Crystal System		Orthorhombic		
Space Group		Pnma		
Lattice Parameters	a	20.36		
	b	20.13		
	c	13.46		
	α = β = γ	90°		
	Volume	5517.21		
Framework Density		1.74	1.75	1.70

**Table 2:** Basic properties of 70# base asphalt.

Physical Parameter	70# Base Asphalt
Penetration/0.1 mm, 25 °C, 100 g, 5 s	69
Softening point/°C	45
Ductility/cm, 15 °C/5 °C	>100
Density/g/cm^3^, 15 °C	1.02

**Table 3:** Characteristics of two zeolites used in the asphalt VOC emission reduction experiment.

Property	Original Zeolite S-Mi	Treated Zeolite SA-MiMe
Si/Al	-	50
Crystallinity (%)	95	94
Average pore diameter (nm)	1.83	2.28

**Table 4:** Comparison between actual VOC reduction efficiency and theoretical simulated adsorption energy.

Time (min)	VOCs	Reduction Efficiency	Adsorption Energy
6.059	n-Hexane	39%	8.2
7.395	Hexane, 2-methyl-	16%	9.2
7.587	Hexane, 3-methyl-	24%	9.3
8.072	Heptane	37%	8.8
5.349	1-Pentene, 2-methyl-	46%	12.1
6.239	1-Hexene	100%	17.4
7.24	Cyclopentene, 1-methyl-	62%	11.5
7.97	1-Heptene	55%	13.3
4.893	Acetone	48%	34.8
6.651	Butanal	100%	31.4
9.914	Toluene	−235%	12.7
11.847	o-Xylene	−431%	5.7

## Data Availability

The original contributions presented in this study are included in the article. Further inquiries can be directed to the corresponding authors.
